# Interventions geared towards strengthening the health system of Namibia through the integration of palliative care

**DOI:** 10.3332/ecancer.2016.653

**Published:** 2016-07-07

**Authors:** Rachel Freeman, Emmanuel BK Luyirika, Eve Namisango, Fatia Kiyange

**Affiliations:** 1 Faculty of Humanities and Social Sciences, Department of Social Work, University of Namibia, PO Box 55150, Rocky Crest Windhoek, 9000, Namibia; 2 African Palliative Care Association, Kampala, PO Box 72518, APCA, 256, Uganda

**Keywords:** Namibia, palliative care, empowerment, service development

## Abstract

The high burden of non-communicable diseases and communicable diseases in Africa characterised by late presentation and diagnosis makes the need for palliative care a priority from the point of diagnosis to death and through bereavement. Palliative care is an intervention that requires a multidisciplinary team to address the multifaceted needs of the patient and family. Thus, its development takes a broad approach that involves engaging all key stakeholders ranging from policy makers, care providers, educators, the public, patients, and families. The main focus of stakeholder engagement should address some core interventions geared towards improving knowledge and awareness, strengthening skills and attitudes about palliative care. These interventions include educating health and allied healthcare professionals on the palliative care-related problems of patients and best practices for care, explaining palliative care as a clinical and holistic discipline and demonstrating its effectiveness, the need to include palliative care into national policies, strategic plans, training curriculums of healthcare professionals and the engagement of patients, families, and communities.

Interventions from a five-year programme that was aimed at strengthening the health system of Namibia through the integration of palliative care for people living with HIV and AIDS and cancer in Namibia are shared. This article illustrates how a country can implement the World Health Organisation’s public health strategy for developing palliative care services, which recommends four pillars: government policy, education, drug availability, and implementation.

## Background

Southern Africa remains the global epicentre of the human immunodeficiency virus (HIV) epidemic. According to UNAIDS statistics, the number of people living with HIV is about 180,000 out of a Namibian population of 2.1 million, with 160,000 adults and 16,000 children up to 14 years of age living with the disease [1]. HIV and AIDS is associated with over 70,000 orphans in Namibia [1].

The rate of new HIV infections, estimated at 8000 in 2011, remains unacceptably high, with young women aged between 15 and 24 accounting for 62% of the new infections. Pregnant women continue to contribute to the HIV pandemic burden and a high prevalence (70%) continues to prevail among sex workers in some suburbs [1].

According to UNAIDS (2012) [1], Namibia is winning the battle to treat those infected with HIV and AIDS, with more than 100,000 people now accessing treatment compared to 10,000 in 2005. However, it should be noted that the antiretroviral treatment (ART) does not eliminate the need for palliative care in HIV. Even patients with sustained access to ART have a high prevalence of physical and psychological symptoms as well as social and spiritual needs that are best addressed through the holistic- and family-based palliative care approach [2, 3]. The need for palliative care in this patient population continues to be critical as good control of physical and psychosocial symptoms is associated with better adherence and thus viral load suppression [4].

Non-communicable diseases (NCDs) (i.e., cardiovascular diseases, mental illnesses, trauma, cancers, chronic respiratory diseases, and diabetes) are also increasingly contributing to the disease burden and need for palliative care. According to the world health organisation (WHO), of the 57 million global deaths in 2008, 36 million (63%) were due to NCDs, with nearly 80% of these deaths occurring in low- and middle-income countries [5]. National level data in Namibia highlights hypertension and diabetes as the first and second causes of disability among adults [6]. Notably, the prevalence of NCDs increased from 6% in 2006 to 8% in 2007. Moreover, data from health information system (HIS) also indicated that heart diseases, hypertension, and strokes were collectively responsible for significant number of a health facility deaths in 2005 [6]. Hospital deaths due to cancers constituted 0.6% between 2005 and 2007 [6]. With such a high disease burden of conditions that can benefit from palliative care [7, 8], it is critical to support efforts geared towards supporting the development of palliative care in the country.

The world health organisation (WHO) defines palliative care as an approach that improves the quality of life of patients (adults and children) and their families who are facing problems associated with life-threatening illness [9]. It prevents and relieves suffering through the early identification, correct assessment, and treatment of pain and other problems, whether physical, psychosocial, or spiritual. Palliative care is explicitly recognised as a human right, under the international human right to health from the International Covenant on Economic, Social and Cultural Rights (ICESCR) Article 12.1 (1996) [9, 10, 11]. There is strong evidence to show that early palliative care improves survival and patient outcomes and for this reason, it should be provided from the point of diagnosis [12]. WHO further emphasises that early palliative care reduces unnecessary hospital admissions and the use of health services [9].

A situation analysis of the status of palliative care in Namibia in 2009 showed very limited availability of palliative care and the presence of home-based care services that were providing inadequate pain and symptom control due to several systemic problems [13]. Palliative care as a concept and discipline is commonly not well understood, and this is the case in Namibia. Findings from the national situation analysis further revealed low level of knowledge for the definition of palliative care [13]. Palliative care is often either seen as synonymous with end of life or death and is mainly provided by a few hospices in Namibia.

Few people comprehend its holistic nature, or know about its effectiveness in improving care outcomes, most importantly the quality of life of patients and their caregivers [2, 13]. Yet palliative care has continued to be on the global and regional health agendas. Palliation is recognised as one of the essential healthcare services required for achieving universal health coverage [14].

In May 2014, the world health assembly (WHA) passed a resolution on strengthening palliative care as a component of comprehensive care throughout the life course [15]. The resolution that is a manifestation of the right to quality care for adults and children with life-limiting illnesses, outlines the responsibilities of WHO member states. These are based on nine key areas: evidence-based palliative care policies; funding and allocation of human resources; basic support to all caregivers, including families, volunteers, and others; education and training at all levels; assessing basic palliative care needs including pain medication requirements; revision of national and local legislation and policies for controlled medicines to improve access; updating national essential medicines lists; fostering partnerships; and implementing and monitoring palliative care actions included in the WHO’s Global Action Plan for the Prevention and Control of NCDs 2013–2020.

The 2014 Global Atlas of Palliative Care of the WHO and Worldwide Hospice and Palliative Care Alliance (WHPCA) [16] indicates that Namibia currently has isolated palliative care provision, an improvement from 2011 where the country was reported to only have some capacity building activities [17]. Initial interventions between 2009 and 2014 through palliative care advocacy, awareness creation and capacity building of healthcare workers and volunteers to deliver palliative care services, have laid a firm foundation upon which Namibia can implement a more structured and systematic integration of palliative care in its health system, in alignment with the WHA resolution. This articles share progress on the interventions undertaken and challenges in the key areas with a view to exchange learning in Africa and globally.

## Methods

Through a five-year country programme (2009–2014) funded by the US Agency for International Development (USAID) in Namibia through USAID’s Regional HIV and AIDS Programme (RHAP) in South Africa, the African Palliative Care Association (APCA), and the Ministry of Health and Social Services (MoHSS) in Namibia implemented interventions geared towards strengthening palliative care for people living with HIV and AIDS. Interventions cut across the key areas of advocacy and awareness creation, capacity building for the provision of palliative care and research. Other local partners, including community home-based care programmes were central to the implementation and success of this programme.

## Advocacy and awareness creation

Various advocacy and awareness creation initiatives were implemented in the country. Palliative care sensitisation for policy makers especially those working with MoHSS, healthcare workers, and volunteers in home-based care (HBC) programmes such as the Catholic AIDS Action (CAA), Hope Hospice and the TONATA Network of People Living with HIV (PLHIV).

Innovative approaches, such as convincing the Government of Namibia to host the 3rd African Palliative Care Conference in 2010, were used to raise the profile of palliative care in the country. One example of a major advocacy and awareness initiative is outlined here. In 2012, annual events, such as the World Hospice and Palliative Care Day, were commemorated in the country to garner and sustain the attention of policy makers, service providers and the general public towards the needs of patients and families who need palliative care services. More specifically a palliative care Education and Awareness week (15–19 October) was organised with leadership from APCA, MoHSS, the National Palliative Care Task Force and USAID Namibia under the global theme ***‘Living to the end: Palliative Care for all in need’****.* This event engaged various stakeholders of palliative care in the country including HIV programmes such as CAA, those in Oncology including the Cancer Association of Namibia, Penduka Drama group, University of Namibia (UNAM), Social Work Department and the School of Nursing, Advanced Community Health Care Services (CoHeNa), Namibian Medical Association, the media – TV One Africa Television Services and NBC Radio and Television Services and the print media (The Republikein). Through their participation in event preparation, these stakeholders obtained a better understanding of the role of palliative care in reducing pain and suffering; improving quality of life and restoring dignity of patients and family members. The education and advocacy campaign was used to influence and increase knowledge and awareness through persuasive communication, geared towards public policy, personal behaviour and attitude change, political, public debate, and legal change.

The five stepped advocacy planning cycle (identifying the issue, setting objectives, identifying targets, assessing resources, and planning) was used in planning this campaign [18, 19]. Activities included extensive media coverage of palliative care in 10 local languages and education sessions targeting various sections of the Namibian population. An Edutainment Day aimed at sensitising and educating students, lecturers (including deans) and counsellors of the University of Namibia on the importance of psychosocial support in palliative care was an innovation in the week. Awareness and education events were guided by the following eight key areas: what is palliative care; why palliative care; symptom and pain management in palliative care; cancer and palliative care; HIV and AIDS and palliative care; antiretroviral therapy and palliative care; key challenges in palliative care and palliative care: a holistic approach.

Other innovative advocacy and awareness activities implemented as part of the palliative care education and awareness week in 2012 included the following:

- Parliamentarian palliative care sensitisation meeting
- Sensitisation of doctors and other clinicians on pain management in palliative care
- Patient’s Edutainment Day attended by 30 patients (adult and paediatric patients) from the cancer wards and 80 other delegates consisting of nurses, social workers, doctors, hospital cleaners, hospital administrative staff and other palliative care stakeholders from non-governmental organisations, private nursing, and hospital practices. The day is now included in the MOHSS national day’s calendar for commemoration annually.
- Advocacy road shows on palliative care.
- Production and mass dissemination of IEC materials on palliative care and pain management.

## Capacity building for palliative care provision

Capacity-building initiatives incorporated basic ongoing mentorship, exchange of learning and clinical placements for especially nurses, enabling them to lead the integration of palliative care in existing health services.

Hospices in neighbouring Zimbabwe such as Island Hospice Services offered a learning environment for palliative care development in Namibia. Training took the form of introductory courses, training of trainers, and more specialist courses at diploma level.

Experiential visits were an innovative approach in programme implementation especially for policy makers. In December 2014, two MoHSS officials in Namibia were supported undertook a policy-related experiential visit in Malawi where they drew important lessons for palliative care integration at the national level.

Tailor-made training programmes were also implemented on effective use of opioids for managing pain for doctors, pharmacists, and other clinicians.

At the institutional level, the capacity of several community-based organisations was built in advocating for and provision of palliative care. These include the following: TONATA network of PLHIV, the Catholic AIDS Action (CAA), Positive Vibes, Advanced Community Health Care Services Namibia (CoHeNa), Aids Care Trust (ACT), Tate Kalunga Mweneka Omukithi Wo ‘Aids’ Moshilongo Shetu (TKMOAMS), Tonata PLWHA Network (TONATA), Hospice of Hope and Sam Nujoma MultiPurpose Centre.

## Research

Several research initiatives were undertaken to contribute to the evidence base for the provision of palliative care in Namibia. They included a street survey of where people prefer to die and a comprehensive review of community home based care services in Namibia.

## Results

Resulting from the interventions in the methods section and some key results from the implementation of the programme are shared.

## Advocacy and awareness creation

Ongoing advocacy-targeting policy makers led to the establishment of a national palliative care taskforce which provided leadership in advocating for policy level changes such as the of essential palliative care medicines. Indeed, by 2014, essential palliative care medicines were included in the Namibia Essential Medicines List (NEMLIST) to ensure drug availability for pain management in Namibia. Morphine that is used to manage moderate to severe pain is now accessible at clinic levels and nurses can do a re-prescription of this medication for pain management.

An experiential visit for policy makers to Malawi enabled them to obtain key lessons that would enhance the further integration of palliative care in Namibia’s health system. Major lessons reported by the policy makers are the role and composition of a National palliative care taskforce, the importance of having a desk officer responsible for palliative care at the national level to ensure proper coordination and accountability, the importance of a national palliative care policy in scaling-up the provision of palliative care service, the role of a national palliative care association in supporting government, the various models of delivering palliative care and the importance of pre-service and in-service education, among others. APCA has observed proactiveness of the MoHSS regarding the integration of palliative care in Namibia’s health system following the visit.

A self-filled evaluation of the visit by the MoHSS officials presented a useful tool for APCA and local stakeholders in Namibia to follow up the implementation of key actions by the MoHSS.

Through ongoing advocacy and awareness creation, the importance of palliative care in HIV prevention, care, and support was highlighted by the five-year programme, resulting in palliative care inclusion in key policy and education documents on HIV and AIDS. The development, translation, and dissemination of information, education and communication (IEC) materials on palliative care into local languages has also contributed to greater awareness and understanding of palliative care and pain management by both community members and those involved in service delivery. Carefully planned advocacy and awareness palliative care activities that included the media have been central to improving knowledge and understanding of the needs of people living with life-threatening and life-limiting illnesses in Namibia. The palliative care awareness and education week in 2012 in particular resulted in a successful advocacy campaign raising awareness on the importance of palliative care. The series of activities in the week had an impact on the community, healthcare service providers, academic institutions, and the general public. Some of this impact is reflected in the following feedback from a cross section of those who benefitted from the education week.

*‘Every day I switched on my television or radio or tuning from the one radio station to the other, then I just hear APCA and Palliative care in different languages. Thank you for all the efforts, you are indeed doing a commendable job, now I know who APCA is and what Palliative care is’.* Listener, Radio 001

As a result of two Afrikaans newspaper articles on palliative care in the Republikein (27 September and 23 October 2012), APCA received eight enquiries from individuals and organisations across the country (Hentiesbay, Keetmanshoop, Oshakati, Windhoek, Swakopmund, Luderitz, Mariental and Karasburg) on how they can come on board in providing palliative care or being trained.

Following a doctor’s education dinner which focused on pain management in palliative care, feedback was received requesting for more such education sessions. Key messages doctors acknowledged to have taken seriously include the fact that morphine is not addictive when used well for pain management and that codeine in children under 12 causes a lot of side effects. Doctors set out to include palliative care in their continuing professional development sessions and in their Medical Congress.

*‘The contents of the presentations were excellent, informative and supported with data. I learnt a lot from the pictures that were shown. This made a long lasting impact of reality. The photos in the presentation left long lasting and important messages on the importance of pain management and treatment. I learnt a lot that pain management and palliative care is a holistic approach which needs to address all other psychosocial issues’* noted one of the doctors.

The Parliamentarian palliative care lunch meeting was used to sensitise parliamentarians on the importance of palliative care. It laid the foundation for discussions on need for a palliative care policy in Namibia, which is currently on the agenda for the MoHSS.

An Educational Drama performance by Penduka Drama Group led to an increase in demand for more sensitisation and training on palliative care from members of the Lower House. APCA was consequently requested by the Deputy Speaker of the Namibian Parliament to engage with the Upper House of Parliament, targeting the Executives of parliament in providing them with training on palliative care.

The student/lecturer Palliative Care Edutainment day held at the University of Namibia led to increased awareness about the need for palliative care from the point of diagnosis to death and the problems of patients living cancer and HIV. It was also appreciated that palliative care improves quality of life and makes it easy for patients to live well despite the life limiting and life threatening conditions. The day further inspired the deans of the Faculty of Humanities and Social Sciences (HSS) and the School of Nursing and Public Health (SONPH) leading to renewed commitment towards the integration of palliative care in academic programmes and interest to develop post-graduate palliative care courses. Indeed palliative care is currently integrated in the Social Work and Nursing programmes of UNAM. The use of student led drama to demonstrate the needs of palliative care patients was an innovation in the week. It is evident from the written evaluations that the drama imparted to the audience’s knowledge.

Some of the feedback from students is captured below:

- ‘I learnt that palliative care and psychosocial support are very important components in a sick person’s life the time they are diagnosed and at end of life. It is a good thing that Namibia has brought the course. The drama was well played and it portrayed every reality of life. Loved everything! The drama properly illustrated the reality on the ground for people suffering from cancer and HIV’.
- ‘I like the fact that the students were applying the knowledge that they gained in palliative care. And they were simply practicing it in a drama form to give a clue to those who don’t have an idea about it. I have learnt that what the students were portraying could be real in life and as a multi-disciplinary team we need to have a responsibility to care for people’.
- ‘Please get social work students involve in palliative care related issues’.
- ‘I learnt through the drama that to have a life threatening illness is not a death sentence’.

The road-based shows and meetings conducted in the Hardap and Omaheke regions, targeting the community at large, church leaders, counsellors, and traditional leaders led to increased awareness about palliative care.

- *‘…we are privileged as Chiefs to hear this Palliative Care for the first time ever since....we have heard about AIDS, TB but this palliative care is new to my ears…’* Senior Chief, Otjombinde Constituency, Omaheke region.
- *‘…This palliative care information you are giving us today is very important ... can CoHeNa bring it to the village… to help us as chiefs to make our people understand this palliative care...’.* Senior Chief for Ovambanderu, Epukiro Constituency.
- *‘…CoHeNa what you have started today… must continue as we will work together to also make the community aware of such an important programme of managing pain...’.* Pastor Elias Kandetu, Gobabis Constituency.
- *‘…this is a new concept that l desire to have on a much wider scale in my constituency…’* Mr Johannes Ortman, Gibeon Constituency.
- *‘… l am blessed ... l was diagnosed with prostate cancer … This pain management that l learnt today is God’s way of answering my plight.... where was CoHeNa in 2009 when l was suffering…?’* Mariental Rural Constituency, Hardap region.

## Capacity building

At the national level, capacity-building activities on palliative care led to the development and piloting of a national training curriculum for palliative care, implemented in the country especially for CHBC programmes. Capacity-building activities targeting academic institutions saw the integration of palliative care in some of these institutions. Following the training of more than 65 social workers working with Government of Namibia and the University of Namibia (UNAM) lecturers, palliative care has been integrated in the Social Work Degree curriculum of UNAM. It has also been integrated in the healthcare workers curriculum of the Namibia Health Training College (NHTC). For the UNAM Social Work program, a training manual has been developed to aid the teaching of palliative care. This is critical for the sustainability of the discipline and services. In addition to basic training, a pool of healthcare workers and volunteers, six nurses in the country, mainly those working with CAA were supported to obtain diploma qualifications in palliative care through Hospice Africa Uganda’s Institute for Palliative Care Training in Africa/Makerere University in Uganda. This played a central role in the successful integration of palliative care within the services of CAA and CHBC in the country. More than 2700 community home-based care providers, including PLHIV, were trained on palliative care. This was a major gain from the programme as Namibia now has a pool of CHBC organisations that are at different levels of integrating palliative care. There has been preliminary integration of palliative care in national HBC guidelines and services.

A detailed account of how three CHBC organisations benefitted from the programme’s capacity-building initiative is shared below:

### i. Tonata network of PLHIV

Tonata, a network of people living with HIV/AIDS in four North Central regions of Omusati, Oshana, Ohangwena and Oshikoto had its organisational and technical capacity in PC strengthened through the grant.

In 2012, TONATA was supported to undertake a strategic organisational review, which resulted into putting in place formal structures for the network with paid secretariat staff. Organisational capacity strengthening in key areas such as governance, strategic planning, leadership and management and financial management was then undertaken.

The network developed its first strategic plan that has since guided its annual planning and activities. As a result of these activities, TONATA’s membership increased from 300 to 600 support groups of PLHIV and from 8000 to more than 9000 PLHIV, captured in its database by December 2014. Through consultation, awareness, and advocacy meetings, TONATA also reached more than 500 provincial and local leaders with HIV and AIDS messages that include palliative care in Oshana, Omusati, Oshikoto, and Ohangwena north-central regions of Namibia. In addition, forums of PLHIV were established throughout these regions leading to an improved representation of PLHIV at provincial level and access to updated information on HIV/AIDS care and support. A total of three forums were established in the regions of Ohangwena, Oshana, Oshikoto, and Omusati, constituting a total representation of more than 60 PLHIV. The pilot forum in Ohangwena has demonstrated strong cooperation and relationship between PLHIV representatives and the Regional council both at the regional and at the constituency level. The change in the attitude of government leaders at regional and constituency level in regards to HIV/AIDS and the contribution of Tonata in Ohangwena is significant. These PLHIV representative forums have also facilitated the expansion of Tonata’s programmes on HIV prevention, care and support, and recognition of PLHIV as equal partners in addressing HIV and AIDS at constituency and Regional level. Improvements in relationship and coordination of Tonata with local leadership including the Regional Aids Coordinating Committee (RACOC) and Constituency Aids Coordinating Committee (CACOC), has also been observed.

APCA also undertook a series of palliative care training workshops for more than 800 of Tonata’s support group members, the staff and members of its board. The training improved their understanding of their right and need to receive comprehensive and quality care and support services. It further enhanced their technical knowledge on palliative care for further advocacy with local leadership, communities and at the national level. Palliative care knowledge at the community level has contributed to the confidence of PLHIV to provide care and support to their counterparts in the communities. It has also contributed to reducing stigma and discrimination. In December 2014, and resulting from an important identified need by Tonata, community-based facilitators, volunteers, community health workers, regional facilitators, peer educators, group coordinators, and community activists benefitted from an introductory training on HIV status disclosure to children. They gained knowledge and skills in order to effectively disclose issues of HIV to children and support other groups and communities to incorporate disclosure of HIV to children in their activities.

*‘This training is so unique from other trainings we have received for the past years, because it tells us how to deal with people in a holistic manner. It has really brought us back to be human. Most people on ARV medication are defaulting due to poor counselling and communication at the health facilities, in the homes and in the communities. Palliative care training addressed effective counselling and communication very well’* one of the participants remarked.

*‘We have never attended such training like this one before. This training has opened our minds and motivated us to work harder to care and support one another’* noted another participant.

In July 2014, the leadership of Tonata, comprising of the programme advisor, programme coordinator, and management committee chairman, was supported to undertake an experiential visit to Uganda to draw lessons from the well-established networks of PLHIV and programmes in Uganda. Among the key lessons, the value of integrated HIV services was at all levels of the health system; the use of a multidisciplinary holistic approach to deliver HIV services; the strong alliance between HIV networks and organisations with government institutions and recognition of equal partnership; the role of male role models in HIV programmes and the importance of expert patient programmes to enhance ownership and sustainability, among others. Tonata is implementing some of these lessons to improve their services and to become a stronger network for PLHIV in Namibia.

### ii. Catholic AIDS Action (CAA)

CAA has adapted a more comprehensive approach to the provision of HIV services for their beneficiaries as a result of USAID/RHAP-supported programme. The programme has strengthened CAA’s capacity to integrate palliative care in its community home-based care model, thereby providing a national model for the provision of home-based palliative care (HBPC). Capacity building of CAA community-based nurses and volunteers was undertaken by APCA through orientation palliative care courses, specialist training at diploma level, clinical placements in Zimbabwe and ongoing support and mentorship throughout project life. By 2012, CAA had registered an expansion of its HBPC service model to all 11 CAA sites from nine of the 13 political regions in Namibia and established a Clinical Desk at management level. This followed the training of 828 community HBPC providers at the sites and provided them with service mentorship through monthly support supervision meetings and home visits. More so community volunteers provided HBPC service to about 7414 (adults and children) clients in their own homes by 2012. The palliative care nurses trained for CAA provided supervision and mentorship to the volunteers on pain management of 189 bedridden or mobile-restricted clients in their home settings and made referrals to health facilities and other stakeholders when required. As a result of CAA’s successful integration of PC in home-based care, the organisation secured a grant from the European Union (EU) to implement HBPC in four regions (Omusati, Zambezi, Omaheke, and Kharas for the period 2011–April 2014. CAA has maintained a dialog with the MoHSS in regards to the integration of its home-based palliative care model into the national HBC service.

### iii. Supporting Positive Vibes (PV)

Positive Vibes was supported to roll out the Positive Health, Dignity and Prevention (PHDP) package through workshops for 101 support groups of PLHIV. The PHDP toolkit was also revised with APCA technical assistance to include pain management. The PHDP toolkit focuses on the role PLHIV can play in enhancing care and prevention efforts. It embeds the prevention of transmission and disease progression into a broader context of human rights, general quality of life and increased participation of PLHIV at all levels of decision making.

The workshops were facilitated by PLHIV, who were given prior training on the use of the toolkit, aimed at strengthening community-based knowledge among people living with HIV on positive living. Training reached 1528 support group members across four regions and significantly improved condom distribution and referrals of PLHIV to access health services.

Other milestones from the programme included the adaptation of training materials for training of health care workers on children’s palliative care.

These were handed over by APCA to USAID Namibia and the MoHSS at the end of the programme in December 2014 to facilitate further adaptation and use in the country.

## Research

An APCA-led review of community home-based care services in Namibia in 2013/2014 revealed that CHBC programmes had adapted a comprehensive model for providing care to PLHIV. The model has incorporated key components that range from prevention to palliative care that includes pain management, end of life care, and bereavement services. The review also revealed that a pool of CHBC providers have been trained on palliative care and have played a key role in its integration in their programmes. The review also outlined some gaps that still remain including inadequate access to opioids especially morphine, as a result of restrictive laws on prescription which is only legally done by doctors. Misconceptions and confusion still exists in Namibia where palliative care is perceived as end of life care. Owing to the huge geographical distances between health facilities in the country, there is a need to train more community health workers who can provide palliative care in their catchment areas.

## Discussion

Programme implementation approaches and interventions from the five-year programme which was aimed at strengthening the integration of palliative care in Namibia demonstrate some milestones at the policy, service provider, community, and academic levels. At the policy level, the establishment of a national palliative care taskforce that has continued to provide leadership in advocating for policy level changes is noted. The inclusion of essential palliative care medicines in Namibia’s Essential Medicines List (NEMLIST) to ensure drug availability for pain management is regarded as a best practice from this programme. This is coupled with morphine being more accessible at clinic levels and nurses being able to do a re-prescription of this medication for pain management.

The use of experiential visits where policy makers from one country are enabled to visit and learn from national level developments of palliative care in another country facilitated peer to peer learning for Namibia policy makers in Malawi. This inter-country learning approach can be more widely used across Africa as countries set out to implement the WHA Resolution on palliative care [15]. The programme identified and set out to build institutional and technical capacities of CHBC programmes in Namibia. Specifically, the involvement of PLIHIV indicated that networks of PLHIV can be strengthened into formal institutions with their voices heard at the national and local level. TONATA did not only benefit from capacity building in palliative care, but also organisational capacity strengthening in key areas such as governance, strategic planning, leadership and management, and financial management. As a result of the programme, TONATA’s membership has increased from 300 to 600 support groups of PLHIV and from 8000 to 9000 PLHIV.

A further lesson is that the involvement of PLHIV and their families in palliative care has long term benefits in relation to HIV programming and the quality of life of individuals, family members and communities. Strengthening the capacity of Catholic AIDS Action (CAA) to integrate palliative care in its community home-based care model, has provided a national model for the provision of home-based palliative care (HBPC) in Namibia, a remarkable lesson from the programme. Palliative care sensitisation, training, mentorship, and opportunities for ongoing learning are important in creating confidence for service provision and training among palliative care service providers and educators. It is demonstrated that advocacy and awareness creation coupled with capacity building of academic institutions leads to the appreciation and actual integration of palliative care in the curricula of such institutions. The integration of palliative care in the Social Work program at the University of Namibia and health care workers curriculum of the Namibia Health Training College (NHTC) is an example from this programme. This programme highlights the role of targeted awareness and education using annual events such as the WHPC Day in influencing change at policy, service provider, academic, and community levels. The role of the media and drama in addressing the challenge of lack of awareness and understanding for palliative care is especially demonstrated. Such communication and engagement with the public addresses issues of personal behaviour and attitude change, political, public debate, or legal change. The role of networking, partnerships, and collaboration is also well demonstrated by this programme where APCA implemented this countrywide programme in collaboration with the MoHSS, CHBC programmes, academic institutions, professional councils, media houses, and donors. Milestones from the programme are attributed to the concerted efforts of such collaboration.

As Namibia continues to plan for the further integration of palliative care in its health system, some gaps and challenges need to be addressed. The lack of a clear country coordinating mechanism and structure for palliative care is an important challenge for the future sustainability of palliative care. This is coupled with the lack of a national palliative care policy and a strategic plans that are critical for the country’s deliberate investment in palliative care services. APCA held initial discussions with the MoHSS about these important issues. The MoHSS recognises palliative care as a cross-cutting issue requiring the involvement of all departments in the MoHSS and stakeholders, including the development of a national policy and defining a national coordination mechanism.

Although CHBC programmes have made good progress in the integration of palliative care, there is still inadequate integration in health facilities leading to a lack of strong palliative care clinical sites. Deliberate efforts to integrate palliative care in public and NGO health facilities need to be undertaken as this would ensure access by PLHIV and other life-limiting illnesses and providing clinical sites for in-service healthcare providers and students being trained on palliative care through universities.

Namibia still has a limited number of prescribers for pain management medicines especially opioids (currently only doctors), although discussions on task sharing and task shifting to allow nurses to prescribe have been initiated. Community Nurses are also not able to re-prescribe stronger pain management drugs such as morphine at household level. More health professionals and CHBC providers need to be trained to deliver palliative care to PLHIV and other chronic illnesses. The integration of palliative care in cancer interventions and the response to other chronic diseases remains limited in Namibia, as more focus was centred on its integration in the HIV response through this five-year programme.

The WHO and WHPCA, in the Global Atlas of Palliative Care at the End of Life (pp.10), identify the diseases that require palliative care for adults and children to include Alzheimer’s and other dementias, cancer, cardiovascular diseases (excluding sudden deaths), cirrhosis of the liver, chronic obstructive pulmonary diseases, diabetes, HIV/AIDS, kidney failure, multiple sclerosis, Parkinson’s disease, rheumatoid arthritis, drug-resistant TB, congenital anomalies (excluding heart abnormalities), blood and immune disorders, meningitis, neurological disorders, and neonatal conditions [16]. As the MoHSS in Namibia recognises palliative care as a cross-cutting issue, further efforts for the development of palliative care need to consider its integration in the response to a wide range of chronic illnesses in children and adults. The provision of palliative care to children, including psychosocial care that includes issues of effective communication and counselling in critical situations such as disclosure of HIV status remains a big challenge to both formal and informal healthcare service providers in Namibia. This calls for the active involvement of MoHSS in its integration into services of PLHIV and other chronic illnesses. Under this programme, training materials on children’s palliative care were developed and it is recommended that they are used for future training in Namibia.

Funding of CHBC programmes remains a challenge for the country. Individual organisations that have integrated palliative care in their CHBC programmes such as CAA have experienced staff turnover (including palliative care trained staff) as a result of declining funding.

There are also challenges with the replenishment of HBC kits in some regions. Vast distances to be covered by CHBC providers, the poor bi-directional referral system, coupled with poverty and alcohol overuse have also reported as challenges for the provision of home-based palliative care.

Male participation in HIV interventions is almost non-existent in Namibia, and this affects the effectiveness of prevention, care, and support efforts at the community level. Male involvement in HIV interventions needs to be scaled up through innovative approaches such as developing awareness and training programmes that target men.

## Conclusion

Initial palliative care interventions in Namibia set out in the five-year programme and shared in this paper have established a firm foundation upon which the country can implement the May 2014 WHA Resolution on palliative care [1]. The leadership and investment of the MoHSS in palliative care coupled with strategic partnerships and collaborations among palliative care advocates, service providers, educators, patients and families and communities will determine the future of palliative care in Namibia and its sustainability. Outcomes from these initial interventions can inform future interventions in Namibia and Africa.

## Conflicts of interest

The authors declare that they have no conflicts of interest.
